# The carotenoid-continuum: carotenoid-based plumage ranges from conspicuous to cryptic and back again

**DOI:** 10.1186/1472-6785-10-13

**Published:** 2010-05-26

**Authors:** Kaspar Delhey, Mark L Roberts, Anne Peters

**Affiliations:** 1 Max Planck Institute for Ornithology, Vogelwarte Radolfzell, Schlossallee 2, D-78315, Radolfzell, Germany; 2 Division of Biology, Imperial College London, Silwood Park Campus, Buckhurst Road, Ascot, Berkshire SL5 7PY, UK

## Abstract

**Background:**

Carotenoids are frequently used by birds to colour their plumage with green, yellow, orange or red hues, and carotenoid-based colours are considered honest signals of quality, although they may have other functions, such as crypsis. It is usually assumed that red through yellow colours have a signalling function while green is cryptic. Here we challenge this notion using the yellow and green colouration of blue tits (*Cyanistes caeruleus*), great tits (*Parus major*) and greenfinches (*Carduelis chloris*) as a model.

**Results:**

The relationship between colouration (chroma, computed using visual sensitivities of conspecifics) and detectability (contrast against natural backgrounds as perceived by conspecifics and avian predators) followed a similar curvilinear pattern for yellow and green plumage with minimum detectability at intermediate levels of carotenoid deposition. Thus, for yellow and green plumage, colours at or close to the point of minimum detectability may aid in crypsis. This may be the case for blue and great tit green and yellow plumage, and greenfinch green plumage, all of which had comparably low levels of detectability, while greenfinch yellow plumage was more chromatic and detectable. As yellow and green blue tit colouration are strongly affected by carotenoid availability during moult, variation in pigment availability between habitats may affect the degree of background-matching or the costliness of producing cryptic plumage.

**Conclusions:**

Increasing carotenoid-deposition in the integument does not always lead to more conspicuous colours. In some cases, such as in blue or great tits, carotenoid deposition may be selected through enhanced background-matching, which in turn suggests that producing cryptic plumage may entail costs. We stress however, that our data do not rule out a signalling function of carotenoid-based plumage in tits. Rather, it shows that alternative functions are plausible and that assuming a signalling function based solely on the deposition of carotenoids in the integument may not be warranted.

## Background

Carotenoids are some of the commonest pigments that confer colour to bird plumage, being responsible for many yellow, orange and red, but also greenish hues [1]. As carotenoids cannot be synthesised *de novo *by birds, they need to be ingested with food [1]. Thus, the extent of carotenoid-based colouration could be a sign of the foraging ability of an individual, which may be indicative of its quality and/or condition [1,2]. Moreover, allocating carotenoids to produce colourful plumage could be traded-off with other important functions such as free-radical scavenging, immune activation, etc. (for a review see [1]). Given the potential of carotenoid-based colouration to convey information about individual quality, it has been often assumed that yellow through red carotenoid-based colours should have a signalling function. For some carotenoid-based plumages the signalling function has been confirmed through careful experimental studies (e.g. [3-7]). In other cases a signalling function has been inferred based on observed correlations between plumage colouration and condition at moult, degree of parasitism, immune-responsiveness, etc. (e.g. [8-13]).

An example of the latter is the yellow ventral colouration of great tits (*Parus major*) and blue tits (*Cyanistes caeruleus*), that are popular model systems for the study of carotenoid-based ornamental plumages. Although yellow colouration in these species (which is based on the deposition of the carotenoids lutein and zeaxanthin obtained directly from the diet [14]) has been intensively studied, a confirmation of its signalling function is still elusive. In general, for both adults and nestlings the intensity of yellow colouration depends on environmental factors at the time of plumage development such as food availability [15,16], carotenoid content of food [17-20], parasitism [21] and pollution levels [22-24]. Therefore in theory yellow colouration could indicate individual quality. In adult tits, some studies suggest that yellower individuals are better at providing food to offspring [25-27], have higher reproductive output [28] or survival [21], although the only available mate choice study found no difference in yellow colouration between preferred and non-preferred male blue tits [29]. In fledglings, evidence in favour of a signalling function is even scarcer, as parents do not seem to discriminate between nestlings based on their yellow colouration [30], and nestling survival is unrelated to their colour [31]. This has led to doubts about the signalling function of yellow colouration in these species [32].

Alternatively, the deposition of yellow carotenoids may be selected to reduce conspicuousness to avian predators. Predation by birds of prey such as the sparrowhawk (*Accipiter nisus*) is an important source of mortality for adult and fledgling tits [33-35], and due to their good colour discrimination abilities (compared to other potential predators such as mammals or reptiles), birds of prey are thus expected to exert strong selection pressure on cryptic colouration (e.g. [36]). Indeed, crypsis was initially proposed as the main function of yellow tit plumage [37], especially in fledglings which are very prone to being depredated [38], but this idea has not received much attention (although see [31,33]). Crypsis has been the usual explanation for the occurrence of the green, carotenoid-based, back colouration in these and other species [39,40], although there is limited evidence that green plumage is cryptic. The main difference between yellow and green plumage is that in the latter yellow carotenoids are deposited on more heavily melanised (grey) feathers ([41] and unpubl. data). For green plumage it seems clear that a certain quantity of yellow carotenoids has to be deposited in order to achieve a more cryptic plumage against natural backgrounds such as green leaves. However, how conspicuousness covaries with colour intensity is unknown (i.e. to what extent does conspicuousness decrease with increasing carotenoid deposition?). Whether carotenoid deposition has the same effect on yellow plumage is unclear.

If yellow plumage functions as a signal we would expect clear differences between green and yellow plumage based on signalling theory [42-44]. Specifically, we would predict that more intense colouration (higher deposition of carotenoids, higher production costs) in yellow plumage should lead to higher detectability compared to green plumage. At the same time, yellow plumage should respond more strongly to the experimental manipulation of carotenoid availability during moult than green colour (e.g. [13]).

Here we use blue and great tit carotenoid-based colouration (yellow and green) as a case study to highlight the effects of carotenoid deposition in the plumage on colouration and detectability. To this end we determined the relationship between colouration and detectability for great tits and blue tits, using data on the natural variation in colouration of adults and fledglings, in combination with experimental manipulation of carotenoid availability during moult. Additionally, in order to show the pattern of variation beyond the range of colouration of blue and great tits, we include reflectance measurements of the greenfinch (*Carduelis chloris*) which possesses more intensely coloured green and yellow plumage patches than the tit species, and where yellow colouration is more sensitive than green colouration to carotenoid-availability during moult [13].

## Methods

### Study area and species

Birds were captured using mist nets in Möggingen, SW Germany (47°45'N, 8°59'E), between March and June from 2005 to 2007. The area consists of a matrix of mixed forest, orchards, gardens, and reed beds. Adult and fledgling great tits and blue tits, and adult greenfinches, were measured (see Reflectance Spectrometry below) directly after capture and then released. Some blue tit measurements were made on fledglings that had been taken into captivity as nestlings (aged 12 days) in May 2006 together with their parents which continued raising them until independence in large outdoor aviaries (under licence from Regierungspräsidium Freiburg 55-8852.15/05 and G-06/05, 35-9185.82/3/339). Their colouration was measured five to six weeks after hatching, when the yellow to greenish-grey juvenile plumage, present until the autumn moult into adult plumage, was fully developed. As the intensity of carotenoid-based colouration is determined early in nestling development [18], food provided in captivity most likely did not affect plumage colouration. Some of these fledglings were subsequently used in an experiment designed to manipulate the availability of carotenoids during moult [45-47]. Fledgling blue tits were fed either a semi-synthetic control diet (11 males and 10 females; no added lutein other than that present in mealworms) or a semi-synthetic enriched diet (9 males and 10 females; with higher protein content and 0.05% of wet weight added lutein) during the post-juvenile moult period (license from Regierungspräsidium Freiburg Nr. Aktenzeichen 55-8852.15/05 and Registriernr. G-06/05, Aktenzeichen 35-9185.82/3/339 respectively). Their plumage reflectance was measured as detailed below the following spring (March 2007).

### Reflectance Spectrometry

Yellow plumage patches measured were the breast of great tits and blue tits and the tail patch of the greenfinch. Their colour is produced by depositing yellow carotenoids (lutein with small amounts of zeaxanthin in the blue tit and great tit, [48,32,14]; and canary xanthophylls A and B in the greenfinch: [48,49]) on white feathers. Olive-green plumage patches (the back in the three species) derive their colour by depositing yellow carotenoids (presumably the same as in yellow plumage) on melanized grey feathers [41]. Despite depositing different carotenoids in the plumage we considered tit and greenfinch carotenoid colouration as a single colour type due to the similarities in absorption profiles of lutein, zeaxanthin and canary xantophylls (all three show absorption maxima between 440-450 nm, [48], see also the similarity in shape of plumage reflectance spectra in Additional File 1). For comparative purposes we also measured the reflectance of the presumably cryptic brown-grey melanin-based back plumage of other species captured at the study site namely: wren, *Troglodytes troglodytes *(N = 3); chiffchaff, *Phylloscopus collybita *(N = 10); blackcap, *Sylvia atricapilla *(N = 66); blackbird, *Turdus merula *(only females, N = 11); and robin, *Erithacus rubecula *(N = 31).

Reflectance spectra were obtained using an Avaspec 2048 spectrometer connected to a deuterium-halogen light source (Avalight-DHS, Avantes, Eerbeek, Netherlands) through a bifurcated fibre optics cable fitted at the end with a plastic cylinder to standardise measuring distance and shield out ambient light. The probe was held perpendicular to the surface of the feathers and illumination and recording angles were both 90°. Reflectance was computed relative to a WS-2 white standard using the program Avasoft 6.2.1. We took a set of five reflectance readings of different predefined and standardized spots in each body part separated by at least 0.5 cm. Reflectance of the breast was measured on the upper breast (i.e. above the belly) taking two readings on either side and one closer to the centre (but avoiding the dark breast stripe). Measurements on the back were taken following the pattern of five points on a die centred on the middle of the back (the rump was not measured). For the greenfinch we also measured tail colour whereby we only measured the reflectance of the yellow patch on the rectrices.

Reflectance of natural backgrounds present in the study area was measured with the same equipment in spring 2008. We measured leaves, small branches and bark of the main trees and shrubs in the area and samples of leaf-litter and grasses, totalizing 1954 reflectance spectra belonging to 26 different types of vegetation (76.7 spectra on average per vegetation type, range: 15-235; see Table 1 in Additional File 2 for a complete list). We separated the sample in the two main types of backgrounds present in the environment, namely green backgrounds (green leaves and grasses) and brown backgrounds (branches, bark and leaf-litter). We computed the average green and brown backgrounds for each species in the sample and then we calculated the overall green and brown averages, weighing the contribution of each species by its relative abundance in the study area.

Reflectance values of plumage and backgrounds between 300 to 700 nm (in 1 nm steps) were imported into custom made spreadsheets for further analysis. Average reflectance spectra of plumage and backgrounds are provided in Additional Files 1 and 2 respectively.

### Colour analyses

Our analyses focused on assessing the relationship between the intensity of carotenoid-based colouration and detectability. Thus, these two components needed to be estimated. The intensity of colouration in carotenoid-based plumage colours is most likely dependent on the quantities of carotenoids deposited in the feathers, and yellow plumage colour (mainly measurements of chroma) correlates positively with carotenoid content in several bird species (great tits [32], greenfinches, [49], American goldfinches *Carduelis tristis *[50]). Thus we (the same as potential receivers interested in assessing a particular bird) used plumage colouration as a proxy for carotenoid content and assumed that plumage with higher chroma contained higher amounts of deposited carotenoids. We defined detectability as the combined chromatic and achromatic visual contrast against natural backgrounds as seen by conspecifics and birds of prey [51][52][53][54], [36]. Birds of prey, such as the sparrowhawk, were chosen as model predators because they are one of the most common and important predators of great and blue tits [33,35,55-59]. At the same time compared to other potential tit predators (mammals or reptiles) birds have a highly sophisticated visual system with good colour discrimination abilities [36,60,61] and thus are expected to exert strong selection pressures on the visual appearance of their prey. Note that we only computed detectability for the specific colours under study and make no claim about the detectability of each species as a whole, which probably also depends on other exposed plumage patches and their size, in combination with behaviour and other traits.

#### Visual modelling

To quantify variation in carotenoid-based colouration and contrast against natural backgrounds we used a physiological model of avian vision [60]. This model, which can be used to compute chromatic and achromatic contrasts between different reflectance spectra, relies on the correct estimation of a series of parameters. Chiefly, data on spectral sensitivities of photoreceptors (including the effects of the ocular media and the effects of oil droplets) is used in combination with the spectrum of ambient light to obtain stimulus quantum catches for each photoreceptor [60]. These are used to compute contrast between two stimuli (reflectance spectra) and contrast depends on the signal-to-noise ratio of the photoreceptors which in turn depends on the relative abundance of the photoreceptors in the retina. Values of contrast will thus depend on the illuminant used, knowledge of the spectral sensitivity of the photoreceptors, and the relative abundance of these photoreceptors.

Colour vision in diurnal birds depends on the four types of single cones, sensitive to very short (VS), short (S), medium (M), and long (L) wavelengths. The spectral sensitivities of the four single cones vary interspecifically, but among birds two main groups can be recognized based on the sensitivity maximum of the VS cone [62,63]: birds with U-type eyes (some passerines, parrots, gulls, trogons), showing peak sensitivity in the near ultraviolet and birds with V-type eyes, such as raptors, with peak VS sensitivity in the violet wavelength range [62]. Therefore, we used generalized spectral cones sensitivities of U- and V-type birds (from Appendix 1 in [63]) to compute cone quantum catches for each cone type, employing formula 1 in Vorobyev et al. [60], with standard daylight (D65) as the illuminant. Changes in illuminant used in the calculations have been shown to affect results but these effects are relatively small due to colour constancy [60,11,64], and are unlikely to affect the results. The choice of generalized U-type cone sensitivities instead of blue tit specific values is unlikely to have affected our results since small differences in sensitivities (peak sensitivities between generalized U-type and blue tit specific sensitivitiess differ by less than 5 nm [63,65]) have been shown to cause minimal differences in contrast [66]. While single cones are used for chromatic tasks in birds, perception of achromatic variation is believed to be mediated by the double cones [67]. We computed double cone quantum catches using the sensitivity spectrum of the double cones of *Leiothrix lutea *[68] and formula 1 in Vorobyev et al. [60] as above.

#### Quantification of carotenoid-based colouration as seen by conspecifics

Single cone quantum catches for U-type eyes were transformed into relative cone quantum catches by dividing each value by the sum of all four. When these are plotted in the avian tetrahedral visual space, each reflectance spectrum is defined as one point, with x, y and z coordinates. Tetrahedral coordinates were computed using the formulas in Kelber et al. [69]. Reflectance measurements from yellow and green plumage patches form more or less discrete cigar shaped clouds in the visual space (see for example figure S2 in [11]). As an estimate of the intensity of carotenoid-based colouration we used Euclidean chroma which is calculated as the Euclidean distance to the achromatic centre (i.e. where all cone types are equally stimulated, [63]). Euclidean chroma (hereafter "chroma") correlates strongly and positively with carotenoid chroma (carotenoid chroma = -2.98 - 19.85*chroma - 26.05*chroma^2^; R^2 ^= 0.93, all p < 0.0001) a variable commonly used to quantify yellow carotenoid-based colouration in birds (computed as (R_700_-R_450_)/R_700_, [70]). Thus similar results are also obtained if we use carotenoid chroma. Note however, that carotenoid-chroma does quickly become saturated at higher carotenoid concentrations (hence the significant quadratic component in the formula above), becoming less sensitive to changes in carotenoid concentrations as shown in simulations [71]. Similar simulations show that higher carotenoid concentrations are needed for Euclidean chroma to become saturated, largely outside the range of colouration of this study (unpubl. data).

#### Quantification of visual contrast as seen by conspecifics and birds of prey

**Figure 1:**
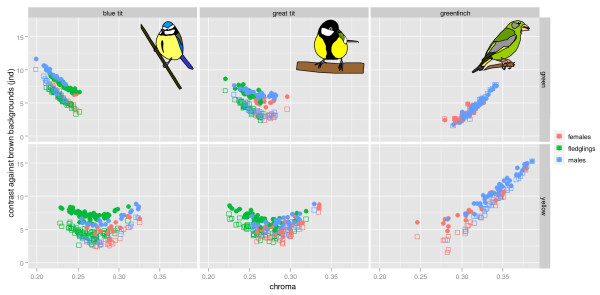
**Relationship between carotenoid-based colouration (chroma) and detectability (visual contrast) against average brown backgrounds**. Upper panels correspond to green and lower panels to yellow plumage of blue tits (left), great tits (centre) and greenfinches (right). Each panel depicts the raw data for males, females and fledglings separately. Filled symbols above correspond to U-type eyes while the open symbols below belong to V-type eyes (note that for the greenfinch back there is much overlap between both eye types).

**Figure 2:**
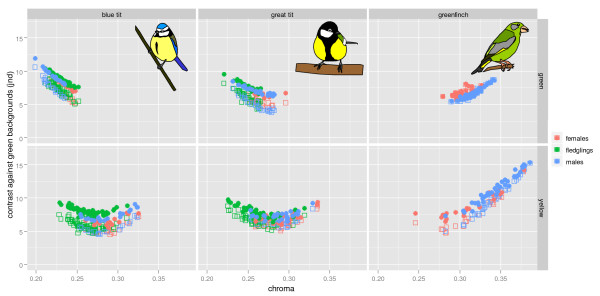
**Relationship between carotenoid-based colouration (chroma) and detectability (visual contrast) against average green backgrounds**. Upper panels correspond to green and lower panels to yellow plumage of blue tits (left), great tits (centre) and greenfinches (right). Each panel depicts the raw data for males, females and fledglings separately. Filled symbols above correspond to U-type eyes while the open symbols below belong to V-type eyes (note that for the greenfinch back there is much overlap between both eye types).

Chromatic visual contrast (ΔS, also called discriminability) between yellow and green plumage patches of each individual and the average green and brown backgrounds was computed using formulas 2, 3, 4 and 8 in Vorobyev et al. [60] which are based on the assumption that discriminability thresholds are set by the signal-to-noise ratio. In order to estimate the signal-to-noise ratio this formula requires knowledge about the proportions of single cones in the retina. Cone proportions are known for the blue tit [72] but not for great tits, greenfinches or putative avian predators such as the sparrowhawk. Since variability in retinal cone proportions can affect the values of contrast [66] we assessed how this parameter affected our results using all know retinal cone proportions [73]. Our analyses (see Additional File 3 for details) indicate that the shape of the relationship between chroma and contrast was similar for all used retinal cone proportions (Figures 1 and 2 in Additional File 3). Therefore we carried out our computations using cone proportions of the blue tit (0.37:0.71:1:1; VS:S:M:L) as representative for U-type eyes and of the peafowl (0.47:0.89:1.04:1) for V-type eyes (as in [74]).

Achromatic contrast (ΔL) was computed in the same way for conspecifics and predators using double cone quantum catches and followed formula 7 in Siddiqui et al. [75] assuming a Weber fraction of 0.05 for the L cone. As birds probably rely on a combination of chromatic and achromatic contrast cues to detect conspecifics or prey [76,54], and in order to reduce the number of statistical tests, we combined chromatic and achromatic contrast in a single measurement following Darst et al. [52], that we termed contrast. Chromatic and achromatic contrasts are measured in 'just noticeable differences' (jnd), where values above 1 jnd are assumed to be discriminable by birds under optimal viewing conditions [60]. As all values of contrast in this study exceeded 1 jnd (see Results) it could be argued that avian predators should always be able to detect these colours, and that variation in contrast is largely irrelevant for crypsis [77]. However, more contrasting colours may be detected from further away. Schaefer et al. [54] showed that crows (*Corvus ossifragus*) detected more contrasting artificial fruit from further away than less contrasting (but contrast >> 1 jnd) fruit, probably due to the differences in chromatic and achromatic contrasts against the background. Similarly, more contrasting lizard and fruit models suffered higher "predation" by birds than less contrasting, but still detectable (i.e. contrast >> 1 jnd) models [51,78]. Moreover, available evidence indicates that contrast seems to scale in a roughly linear fashion with behavioural measurements of perception. Cazetta et al. [78] found that removal rate of highly contrasting artificial fruit by birds was linearly correlated with chromatic contrast up to high values of contrast (> 40 jnd) beyond which the relationship flattened out. Similarly, but at the low end of contrast values (0-4 jnd), the rate of rejection of artificial eggs from chaffinch (*Fringilla coelebs*) nests followed a linear function of their chromatic contrast [79]. Thus, it is likely that variation in contrast above the 1 jnd threshold contributes to the variation in overall conspicuousness. Finally, a cryptic colour may not necessarily need to match an average background perfectly but rather to be within the levels of contrast found among elements of the background (e.g. [80]). We tried to include this aspect by comparing the contrast between plumage and average backgrounds with the levels of contrast among background elements. In order to do so we computed the contrast between each background (i.e. each plant-type-specific brown and green average) and the global green and brown background averages.

## Results

**Figure 3:**
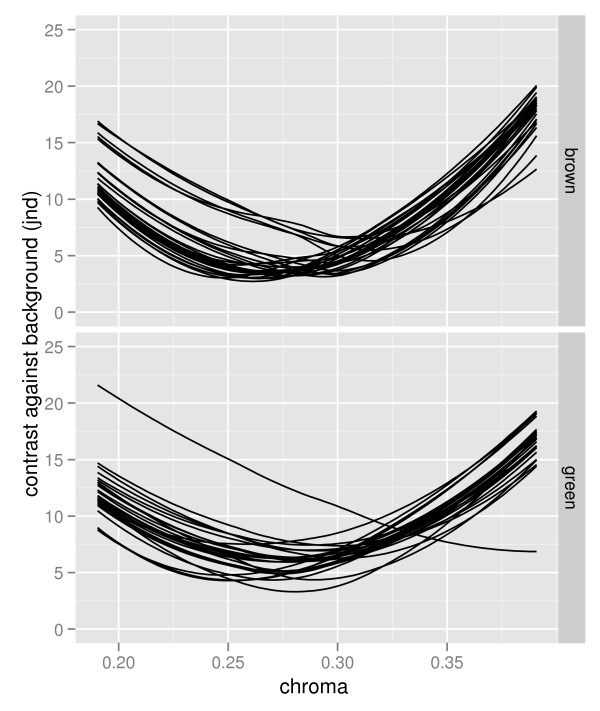
**Relationship between carotenoid-based colouration (chroma) and detectability (visual contrast) against all backgrounds**. Detectability was computed against average brown (above) and green (below) backgrounds computed for each plant species separately using parameters for V-type eyes (results for U-type eyes are very similar) pooling all three species and both green and yellow plumage patches. Depicted are LOWESS regression lines (see [92] for a detailed explanation) for each plant type. Figures depicting this relationship for each background type separately are found in Additional File 4.

Taking all three species together the relationship between chroma and detectability followed a curvilinear pattern with minimum detectability at intermediate values of chroma for both yellow and green plumage (Figures 1 and 2). This relationship was very similar for U- versus V-type eyes (although in general contrast was higher for U-type eyes with blue tit cone proportions than for V-type eyes with peafowl cone proportions [74], but see Additional File 3 for an exception) and for both main types of average natural backgrounds. Roughly similar curvilinear relationships were obtained if instead of the global averages we used the average green and brown backgrounds of each plant species separately (Figure 3 and Additional File 4).

**Figure 4:**
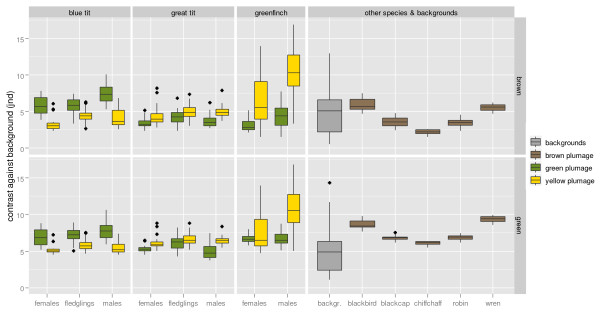
**Detectability of carotenoid-based plumage against natural backgrounds compared with other cryptic plumages and within-background contrast**. Detectability was computed against average brown (above) and green (below) backgrounds. Within-background contrast depicts the level of contrast between different types of backgrounds (see Additional File 2) and the global brown (above) or green (below) averages. Boxplots depict maximum and minimum, 75 and 25 quantiles and median. Dots represent outliers.

For yellow and green plumage patches, as chroma increased (due to increased carotenoid deposition) their detectability to both conspecifics and potential predators decreased until a minimum was reached at intermediate levels of carotenoid deposition. Beyond this point further increase in chroma led to higher detectability. The three species studied here occupied different parts of this 'carotenoid continuum', with blue tits having the lowest, great tit intermediate and greenfinches highest levels of carotenoid deposition. Within each species the range of carotenoid variation was further partitioned by sex and/or age class. This information is detailed in Table 1, Figures 1, 2 and 4 and summarized below.

**Table 1:** Sex and age-related differences in carotenoid-based coloration (chroma) and detectability (visual contrast in jnd).

			**males**			**females**			**fledglings**		
					
			**mean**	**SE**	**N**	**mean**	**SE**	**N**	**mean**	**SE**	**N**
					
blue tit	yellow breast	chroma	0.287^a^	0.005	20	0.288^a^	0.004	17	0.263^b^	0.002	71
		Contrast_U_G	7.42^a^	0.17	20	6.57^b^	0.15	17	8.02^c^	0.06	71
		Contrast _V_G	5.53^ab^	0.20	20	5.26^b^	0.17	17	5.81^a^	0.07	71
		Contrast _U_B	6.72^a^	0.23	20	5.34^b^	0.22	17	7.27^c^	0.07	71
		Contrast _V_B	4.26^a^	0.31	20	3.39^b^	0.27	17	4.42^a^	0.09	71
	green back	chroma	0.218^a^	0.002	20	0.232^b^	0.002	17	0.228^b^	0.001	54
		Contrast _U_G	9.58^a^	0.22	20	8.27^b^	0.22	17	8.99^c^	0.10	54
		Contrast _V_G	7.75^a^	0.27	20	6.91^b^	0.29	17	7.23^b^	0.11	54
		Contrast _U_B	9.47^a^	0.23	20	7.66^b^	0.21	17	8.08^b^	0.11	54
		Contrast _V_B	7.34^a^	0.29	20	5.90^b^	0.30	17	5.79^b^	0.13	54
											
great tit	yellow breast	chroma	0.287^a^	0.003	27	0.289^a^	0.004	23	0.262^b^	0.003	43
		Contrast _U_G	7.40^a^	0.11	27	7.00^b^	0.17	23	7.98^c^	0.11	43
		Contrast _V_G	6.44^ab^	0.11	27	6.18^a^	0.19	23	6.61^b^	0.12	43
		Contrast _U_B	6.28^a^	0.15	27	5.57^b^	0.24	23	6.84^c^	0.13	43
		Contrast _V_B	4.99^ab^	0.16	27	4.35^a^	0.28	23	4.97^b^	0.15	43
	green back	chroma	0.261^a^	0.003	27	0.264^a^	0.002	23	0.248^b^	0.001	43
		Contrast _U_G	7.09^a^	0.11	27	6.76^b^	0.08	23	7.78^c^	0.09	43
		Contrast _V_G	4.99^a^	0.19	27	5.34^a^	0.10	23	6.13^b^	0.13	43
		Contrast _U_B	6.47^a^	0.09	27	5.55^b^	0.11	23	6.59^a^	0.11	43
		Contrast _V_B	3.75^a^	0.18	27	3.42^a^	0.13	23	4.26^c^	0.14	43
											
greenfinch	yellow tail patch	chroma	0.344^a^	0.003	41	0.312^b^	0.007	20			
		Contrast _U_G	11.42^a^	0.33	41	8.94^b^	0.44	20			
		Contrast _V_G	10.21^a^	0.40	41	7.45^b^	0.55	20			
		Contrast _U_B	11.20^a^	0.36	41	8.24^b^	0.53	20			
		Contrast _V_B	9.88^a^	0.45	41	6.31^b^	0.72	20			
	green back	chroma	0.316^a^	0.002	41	0.302^b^	0.002	20			
		Contrast _U_G	6.79^a^	0.13	41	6.90^a^	0.14	20			
		Contrast _V_G	6.69^a^	0.13	41	6.77^a^	0.13	20			
		Contrast _U_B	4.56^a^	0.22	41	3.19^b^	0.22	20			
		Contrast _V_B	4.53^a^	0.23	41	3.19^b^	0.21	20			

Values of contrast are modelled for U- and V-type eyes (represented by U and V respectively) and for average green and brown backgrounds (G and B respectively). Presented are means, standard errors (SE) and sample sizes (N). Means with the same superscript letter are not statistically different (based on t-tests, p > 0.05).

In blue and great tits, yellow breast colouration was more intense in adults than in fledglings, while there was little sexual dichromatism in adults. Conversely, sexual dichromatism in yellow tail colouration was marked in greenfinches where males had more intense colouration than females. Chromatic differences may or may not lead to differences in detectability depending on the position along the carotenoid continuum (Figures 1 and 2). Thus, while fledgling tits differed significantly from adults in their colouration, the difference in detectability was small (0 - 1.9 jnds depending on the background and eye type). Sex differences in detectability for both tit species were also small (0.25 - 1.3 jnd). In greenfinches on the other hand, the more elaborate ornamentation of males resulted in the increased detectability of this plumage patch compared to females (difference = 2.5 - 3.5 jnds).

The green back colouration of fledgling great tits was less intense than in adults, which showed little sexual dichromatism. For blue tits on the other hand, fledglings showed more intense back colouration than males, and slightly less than females. Unlike great tits, adult blue tits showed sexually dichromatic green back colouration, females being more chromatic. Green back colouration was moderately sexually dichromatic for greenfinches, males being more chromatic than females. Adult female blue tits and fledglings differed little in detectability (-0.7 - 0.1 jnd) which was lower than that of adult males (0.5 - 1.8 jnd), while adult male and female great tits were in general less contrasting than fledglings although differences were small (-0.02 - 1.3 jnd). Among greenfinches, the small differences in colouration between males and females led to small differences in detectability, but only against brown backgrounds (males being more detectable than females, brown backgrounds, 1.3 jnd; green backgrounds, -0.08 - -0.012 jnd).

Average levels of contrast between carotenoid-based tit coloration and background were inside the range of within-background contrast (Figure 4). Average levels of contrast of tit carotenoid-based plumage are comparable to those of the melanin-based back plumage of other local species (Figure 4), some of which are typically considered highly cryptic (e.g. wren or chiffchaff).

Carotenoid availability during moult strongly affected yellow and green plumage in male and female blue tits (Table 2). Birds fed carotenoid-enriched diet had higher chroma than control birds. Control birds were considerably less chromatic than wild-caught birds (compare values in Tables 1 and 2) suggesting that the amount of carotenoids available to them was lower than local carotenoid availability in the wild. Experimental effects on carotenoid-based colouration led to differences in detectability in blue tit yellow and green plumage, in that carotenoid supplemented birds had lower detectability, this effect being particularly strong for the green back plumage (yellow = -0.3 - -1.8 jnd; green = -2.1 - -3.5 jnd).

**Table 2:** Effects of the manipulation of dietary carotenoid availability on yellow and green plumage coloration (chroma) and detectability (visual contrast in jnd) in blue tits.

		**males**						**females**						**ANOVA**			
						
		**control**			**carotenoid suppl**.			**control**			**carotenoid suppl**.			**sex**	**treat**.	**s × t**	**adj. R^2^**
						
		**mean**	**SE**	**N**	**mean**	**SE**	**N**	**mean**	**SE**	**N**	**mean**	**SE**	**N**				
									
yellow breast	chroma	0.238	0.003	11	0.281	0.003	9	0.239	0.002	10	0.273	0.004	10	NS	**	NS	0.77
	Contrast _U_G	8.59	0.21	11	7.86	0.08	9	8.48	0.14	10	7.46	0.13	10	NS	**	NS	0.45
	Contrast _V_G	6.93	0.24	11	5.08	0.07	9	6.91	0.15	10	5.18	0.16	10	NS	**	NS	0.72
	Contrast _U_B	7.68	0.22	11	7.37	0.13	9	7.44	0.16	10	6.68	0.13	10	**	**	NS	0.3
	Contrast _V_B	5.50	0.27	11	3.95	0.17	9	5.33	0.17	10	3.67	0.18	10	NS	**	NS	0.6
																	
green back	chroma	0.201	0.002	11	0.224	0.002	9	0.210	0.002	10	0.237	0.002	10	**	**	NS	0.85
	Contrast _U_G	11.63	0.28	11	9.30	0.16	9	10.60	0.13	10	8.25	0.19	10	**	**	NS	0.8
	Contrast _V_G	10.09	0.31	11	6.57	0.25	9	9.27	0.18	10	5.80	0.24	10	**	**	NS	0.83
	Contrast _U_B	11.41	0.30	11	9.27	0.19	9	10.02	0.12	10	7.90	0.20	10	**	**	NS	0.78
	Contrast _V_B	9.60	0.33	11	6.20	0.27	9	8.36	0.17	10	4.93	0.27	10	**	**	NS	0.82

Values of contrast are modelled for U- and V-type eyes (represented by U and V respectively) and for average green and brown backgrounds (G and B respectively). Presented are means, standard errors (SE), sample sizes (N) and significance values for each factor (sex, treatment and their interaction) in ANOVAs (**p < 0.01; NS = not significant, p > 0.05).

## Discussion

We found more similarities than differences between yellow and green plumage. For both types of plumage the relationship between carotenoid-based colouration and detectability followed a comparable curvilinear pattern with minimum detectability at intermediate levels of carotenoid deposition (pooling the data from the three species, Figures 1 and 2). While in general yellow plumage patches were more chromatic than green plumage, both had similar levels of low detectability (contrast against natural backgrounds) in tits. Furthermore, our experiments indicate that, at least for the blue tit, the yellow breast colouration and the green back colouration are both strongly affected by carotenoid availability during moult (Table 2). These patterns were very similar for all types of measured natural backgrounds (Figure 3) and for both types of avian visual systems (Figures 1 and 2).

### Honest yellow signals?

Individual variation in the ability to obtain and metabolise carotenoids is assumed to enforce the honesty of carotenoid-based signalling, thus maintaining the correlation between individual variation in colouration and quality [2]. The curvilinear relationship between carotenoid deposition and conspicuousness indicates that only for colours with levels of carotenoid deposition beyond the point of minimum detectability (Figures 1 and 2) is it likely that increasing carotenoid-deposition will be (mainly) favoured by selection through signalling. This is best exemplified by the yellow tail patch of the male greenfinch which has been demonstrated to fulfill the requirement of heightened condition-dependence of a signalling (indicator) trait since it responds disproportionately to the dietary availability of carotenoids compared to other carotenoid-based plumage patches [13].

Similarly, yellow carotenoid-based colouration in blue and great tits is generally assumed to act as a signal of quality both in adults and fledglings (see Introduction). Indeed, our results (Table 2) and published information [15,17,70,19,27] show that yellow has the potential to convey information about carotenoid availability and hence foraging proficiency or quality. However, sensitivity of the yellow colouration to carotenoid availability is not much greater than the (apparently cryptic [40]) olive-green back colour (Table 2), contrary to the central assumption of greater condition-dependence of signalling traits [44,13]. Likewise, average detectability of yellow and green plumage was similar and within or below the range of what are considered classical examples of cryptic brownish plumages (Figure 4). Moreover, for all these plumage patches contrast fell within the range of contrast found among background elements (Figure 4) suggesting that contrast between plumage and backgrounds could be hidden within the contrast of the background itself (also see [80]). While low detectability does not preclude a signalling function, it does imply that it is not necessary to invoke signalling in order to explain carotenoid deposition in these feathers.

### Optimised for crypsis?

We hypothesise that selection for crypsis is the most likely driving force behind increasing carotenoid deposition at concentrations below the trough of minimum detectability, since increasing carotenoid-deposition will lead to reduced detectability. Selection for reduced conspicuousness, everything else being equal, should cause plumage colouration to converge towards the optimally cryptic phenotype (i.e. the trough of minimum detectability in Figures 1 and 2). Why then, do not all individuals have optimally cryptic carotenoid-based plumage? Or, in other words, what constrains the development of optimally cryptic carotenoid-based colouration? One possibility is that intraspecific variation in colouration does not lead to significant variation in detectability to predators. This may be the case for the yellow colouration of blue and great tits since their average is close to the optimum and the correlation between colouration and detectability not very strong (Figs 1 and 2). Indeed, our experimental manipulation of carotenoid availability during moult in blue tits had strong effects on chroma for both yellow and green plumage but only in the latter did it translate into large differences in detectability (Table 2). Accordingly, Fitze & Tschirren [31] did not find any relationship between natural variation in yellow ventral colouration of great tit fledglings and survival or recruitment, which could have been expected if coloration correlates with predation risk. More extreme variation in colouration through experimental reddening of the ventral plumage, on the other hand, led to a (slightly) increased risk of capture by sparrowhawks [33]. Most likely, the relationship between predation risk and contrast of carotenoid-based plumage colouration will vary markedly in space and time and will also depend on other factors such as the contrast of other colour patches and the behaviour of the birds. One additional factor however, that will constrain the acquisition of optimally cryptic carotenoid-based plumage more directly is the availability of carotenoids which ultimately will determine the colour of the birds.

Carotenoid availability can vary strongly between habitats and this can affect the expression of carotenoid-based colouration. Variation in carotenoid availability has usually been considered in the context of costly carotenoid-based ornamental colours [81-83] but rarely with respect to the development of cryptic carotenoid-based colours (but see [37,84]), perhaps because the expression of naturally selected traits is often (erroneously) assumed to be condition-independent [44]. If in some habitats carotenoid availability is limiting, or if carotenoids are allocated to other important functions such as immune defense [85,86], there may be real constraints involved in producing optimally cryptic carotenoid-based plumage. Costs of developing cryptic plumage are usually neglected [87], but may be an important component in preventing an optimal match between organisms and their backgrounds. Our experiment indicates that both yellow and green blue tit plumage respond strongly to the availability of dietary carotenoids during moult. Thus, lack or excess of carotenoids in the diet may lead to considerable variation in the costs of producing an optimally cryptic plumage or to poor background matching.

Alternatively, environmental variation in carotenoid availability may not necessarily cause mismatches between natural backgrounds and cryptic colouration. Carotenoid-availability, carotenoid-based colouration, and colouration of the natural backgrounds could covary in such a way that more intensely coloured birds are equally cryptic in carotenoid-rich habitats, as less intensely coloured birds in carotenoid-poor habitats [37]. Still untested, this is a plausible hypothesis since the optimal level of carotenoid colouration needed to achieve minimum detectability varied with plant species over a broad range (Figure 3) and differences in the floristic composition and structure of forests are known to affect carotenoid availability [37,88-90]. On a smaller scale, moderate differences between species or individuals in their carotenoid-based colours may lead to similar levels of detectability if birds differ adaptively in their choice of microhabitats. Indeed, differences in foraging microhabitats between great and blue tits may be in part responsible for the differences in back colouration between these two species [40].

## Conclusions

Knowledge on the relationship between colouration and detectability may provide insights into the function of animal coloration. Curvilinear relationships such as shown here for yellow carotenoid pigments may also be found for other types of pigmentary colours (e.g. melanins, psittacofulvins, etc). The shape of the relationship between detectability and colouration may be helpful to determine which level of colour intensity leads to maximum crypsis or conspicuousness. This could, in turn, reveal potential costs and constraints in the development of cryptic or conspicuous plumage. We stress, however, that this is only a first step towards understanding of the function of colours, and careful studies which take into account the detectability of the whole suite of plumage patches and behaviour, are needed to confirm our hypotheses. Meanwhile, and in the same way as we now know that yellow colouration is not always carotenoid-based [91]; our data suggest that assuming all yellow carotenoid-based colours to have a signalling function may be equally flawed.

## Authors' contributions

KD: designed study, collected and analyzed data, wrote the manuscript, MLR: carried out the carotenoid-supplementation experiment, collected data, AP: participated in the design of the study and writing the manuscript. All authors read and approved the final manuscript.

## Supplementary Material

Additional file 1 **Plumage Reflectance Spectra of blue tits (Figure 1), great tits (Figure 2) and greenfinches (Figure 3)**. Depicted are mean reflectance spectra for males, females and fledglings and treatment groups (blue tit only). Click here for file

Additional file 2 **List of common plants in the study area (Table 1, including relative abundance and number of collected reflectance spectra (N)) and their reflectance spectra (Figure 1).** Depicted are mean reflectance spectra for brown (branches, twigs, leaf-litter) and green (leaves) backgrounds. Red lines represent mean spectra for each plant type separately and blue lines the overall brown and green averages taking into account the abundance of each plant type. Click here for file

Additional file 3 Effects of inter-specific variation in cone proportions Click here for file

Additional file 4 Figures representing the relationship between chroma and detectability for each type of background depicting raw data points. Click here for file

## References

[B1] McGraw K Mechanics of Carotenoid-Based Coloration Bird Coloration. Mechanisms and Measurements 2006 1 Cambridge, Massachusetts: Harvard University Press 177 242

[B2] Hill GE Environmental Regulation of Ornamental Coloration Bird Coloration. Mechanisms and Measurements 2006 1 Cambridge, Massachusetts: Harvard University Press 507 560

[B3] Pryke SR Lawes MJ Andersson S Agonistic carotenoid signalling in male red-collared widowbirds: aggression related to the colour signal of both the territory owner and model intruder Animal Behaviour 2001 62 695 704 10.1006/anbe.2001.1804

[B4] Sundberg J Female yellowhammers (*Emberiza citrinella*) prefer yellower males: a laboratory experiment Behavioral Ecology and Sociobiology 1995 37 275 282 10.1007/BF00177407

[B5] Sundberg J Dixon A Old, colourful male yellowhammers, *Emberiza citrinella*, benefit from extra-pair copulations Animal Behaviour 1996 52 113 122 10.1006/anbe.1996.0157

[B6] Hill GE Female house finches prefer colourful males: sexual selection for a condition-dependent trait Animal Behaviour 1990 40 563 572 10.1016/S0003-3472(05)80537-8

[B7] Pryke SR Andersson S Carotenoid-based status signalling in red-shouldered widowbirds (*Euplectes axillaris*): epaulet size and redness affect captive and territorial competition Behavioral Ecology and Sociobiology 2003 53 393 401

[B8] Figuerola J Muñoz E Gutiérrez R Ferrer D Blood parasites, leucocytes and plumage brightness in the Cirl Bunting, *Emberiza cirlus* Functional Ecology 1999 13 594 601 10.1046/j.1365-2435.1999.00354.x

[B9] Fenoglio S Cucco M Malacarne G Bill colour and body condition in the Moorhen *Gallinula chloropus* Bird Study 2002 49 89 92 10.1080/00063650209461249

[B10] Negro JJ Grande JM Tella JL Garrido J Hornero D Donázar JA Sanchez-Zapata JA Benítez JR Barcell M Coprophagy: An unusual source of essential carotenoids Nature 2002 416 807 808 10.1038/416807a 11976670

[B11] Delhey K Peters A Quantifying variability of avian colours: are signalling traits more variable? PLoS ONE 2008 3 10.1371/journal.pone.0001689 18301766 PMC2253496

[B12] Lopez G Figuerola J Soriguer R Carotenoid-based masks in the European Goldfinch *Carduelis carduelis *reflect different information in males and females Ardea 2008 96 233 242

[B13] Peters A Delhey K Andersson S Noordwijk HV Förschler MI Condition-dependence of multiple carotenoid-based plumage traits: an experimental study Functional Ecology 2008 22 831 839 10.1111/j.1365-2435.2008.01437.x

[B14] Partali V Liaaen-Jensen S Slagsvold T Lifjeld J Carotenoids in food chain studies II. The food chain of *Parus *spp. monitored by carotenoid analysis Comparative Biochemistry and Physiology Part B: Biochemistry and Molecular Biology 1987 87 885 888 10.1016/0305-0491(87)90408-1

[B15] Hõrak P Vellau H Ots I Møller AP Growth conditions affect carotenoid-based plumage coloration of great tit nestlings Naturwissenschaften 2000 87 460 464 10.1007/s001140050759 11129946

[B16] Jacot A Kempenaers B Effects of nestling condition on UV plumage traits in blue tits: an experimental approach Behav Ecol 2007 18 34 40 10.1093/beheco/arl054

[B17] Tschirren B Fitze PS Richner H Proximate mechanisms of variation in the carotenoid-based plumage coloration of nestling great tits (*Parus major *L.) Journal of Evolutionary Biology 2003 16 91 100 10.1046/j.1420-9101.2003.00483.x 14635884

[B18] Fitze PS Tschirren B Richner H Carotenoid-based colour expression is determined early in nestling life Oecologia 2003 137 148 152 10.1007/s00442-003-1323-3 12856205

[B19] Hadfield JD Owens IPF Strong environmental determination of a carotenoid-based plumage trait is not mediated by carotenoid availability Journal of Evolutionary Biology 2006 19 1104 1114 10.1111/j.1420-9101.2006.01095.x 16780511

[B20] Fitze PS Tschirren B Gasparini J Richner H Carotenoid-Based Plumage Colors and Immune Function: Is There a Trade-Off for Rare Carotenoids? The American Naturalist 2007 169 S137 S144 10.1086/510094 19426088

[B21] Hõrak P Ots I Vellau H Spottiswoode C Pape Møller A Carotenoid-based plumage coloration reflects hemoparasite infection and local survival in breeding great tits Oecologia 2001 126 166 173 10.1007/s004420000513 28547614

[B22] Eeva T Lehikoinen E Ronkä M Air pollution fades the plumage of the Great Tit Functional Ecology 1998 12 607 612 10.1046/j.1365-2435.1998.00221.x

[B23] Eeva T Sillanpää S Salminen J Nikkinen L Tuominen A Toivonen E Pihlaja K Lehikoinen E Environmental Pollution Affects the Plumage Color of Great Tit Nestlings through Carotenoid Availability EcoHealth 2008 5 328 337 10.1007/s10393-008-0184-y 18704585

[B24] Dauwe T Eens M Melanin- and carotenoid-dependent signals of great tits (*Parus major*) relate differently to metal pollution Naturwissenschaften 2008 95 969 973 10.1007/s00114-008-0400-1 18506415

[B25] Senar JC Figuerola J Pascual J Brighter Yellow Blue Tits Make Better Parents Proceedings: Biological Sciences 2002 269 257 261 10.1098/rspb.2001.1882 PMC1690890 11839194

[B26] Johnsen A Delhey K Schlicht E Peters A Kempenaers B Male sexual attractiveness and parental effort in blue tits: a test of the differential allocation hypothesis Animal Behaviour 2005 70 877 888 10.1016/j.anbehav.2005.01.005

[B27] Isaksson C Uller T Andersson S Parental effects on carotenoid-based plumage coloration in nestling great tits, Parus major Behavioral Ecology and Sociobiology 2006 60 556 562 10.1007/s00265-006-0200-6

[B28] Doutrelant C Gregoire A Grnac N Gomez D Lambrechts M Perret P Female coloration indicates female reproductive capacity in blue tits Journal of Evolutionary Biology 2008 21 226 233 18034808 10.1111/j.1420-9101.2007.01451.x

[B29] Hunt S Bennett ATD Cuthill IC Griffiths R Blue tits are ultraviolet tits Proceedings of the Royal Society B: Biological Sciences 1998 265 451 10.1098/rspb.1998.0316

[B30] Tschirren B Fitze PS Richner H Carotenoid-based nestling colouration and parental favouritism in the great tit Oecologia 2005 143 477 482 10.1007/s00442-004-1812-z 15678330

[B31] Fitze PS Tschirren B No evidence for survival selection on carotenoid-based nestling coloration in great tits (*Parus major*) Journal of Evolutionary Biology 2006 19 618 624 10.1111/j.1420-9101.2005.01008.x 16599936

[B32] Isaksson C Örnborg J Prager M Andersson S Sex and age differences in reflectance and biochemistry of carotenoid-based colour variation in the great tit *Parus major* Biological Journal of the Linnean Society 2008 95 758 765 10.1111/j.1095-8312.2008.01033.x

[B33] Götmark F Olsson J Artificial colour mutation: do red-painted great tits experience increased or decreased predation? Animal Behaviour 1997 53 83 91 10.1006/anbe.1996.0280

[B34] Geer T The selection of tits *Parus *spp. by sparrowhawks *Accipiter nisus* Ibis 1982 124 159 167 10.1111/j.1474-919X.1982.tb03754.x

[B35] Perrins C Geer T The effect of sparrowhawks on tit populations Ardea 1980 68 133 142

[B36] Stuart-Fox D Moussalli A Whiting MJ Predator-specific camouflage in chameleons Biology Letters 2008 4 326 329 10.1098/rsbl.2008.0173 18492645 PMC2610148

[B37] Slagsvold T Lifjeld JT Variation in plumage colour of the Great tit *Parus major *in relation to habitat, season and food Journal of Zoology Series A 1985 206 321 328

[B38] Naef-Daenzer B Widmer F Nuber M Differential Post-Fledging Survival of Great and Coal Tits in Relation to Their Condition and Fledging Date Journal of Animal Ecology 2001 70 730 738 10.1046/j.0021-8790.2001.00533.x

[B39] Dyck J Determination of plumage colours, feather pigments and -structures by means of reflection spectrophotometry Dansk ornithologisk forening 1966 60 49 76

[B40] Bursell J Dyck J Background matching and evolution of cryptic colours of selected passerines in deciduous woodlands Lundiana 2003 4 51 59

[B41] Lucas AM Stettenheim PR Avian Anatomy Integument Part II 1972 Washington DC: US Department of Agriculture

[B42] Endler JA Signals, Signal Conditions, and the Direction of Evolution The American Naturalist 1992 139 S125 S153 10.1086/285308

[B43] Endler JA Some general comments on the evolution and design of animal communication systems Philosophical Transactions: Biological Sciences 1993 340 215 225 10.1098/rstb.1993.0060 8101656

[B44] Cotton S Fowler K Pomiankowski A Review Paper. Do Sexual Ornaments Demonstrate Heightened Condition-Dependent Expression as Predicted by the Handicap Hypothesis? Proceedings: Biological Sciences 2004 271 771 783 10.1098/rspb.2004.2688 PMC1691662 15255094

[B45] Kurvers RH Roberts ML McWilliams SR Peters A Experimental manipulation of testosterone and condition during molt affects activity and vocalizations of male blue tits Hormones and Behavior 2008 54 263 269 10.1016/j.yhbeh.2008.03.011 18468606

[B46] Roberts ML Ras E Peters A Testosterone increases UV reflectance of sexually selected crown plumage in male blue tits Behav Ecol 2009 20 535 541 10.1093/beheco/arp028

[B47] Roberts M Peters A Is testosterone immunosuppressive in a condition-dependent manner? An experimental test in blue tits Journal of Experimental Biology 2009 212 1811 1818 10.1242/jeb.031047 19482998

[B48] Stradi R Celentano G Rossi E Rovati G Pastore M Carotenoids in bird plumage--I. The carotenoid pattern in a series of palearctic carduelinae Comparative Biochemistry and Physiology Part B: Biochemistry and Molecular Biology 1995 110 131 143 10.1016/0305-0491(94)00136-I

[B49] Saks L McGraw K Horak P How Feather Colour Reflects Its Carotenoid Content Functional Ecology 2003 17 555 561 10.1046/j.1365-2435.2003.00765.x

[B50] Shawkey MD Hill GE McGraw KJ Hood WR Huggins K An experimental test of the contributions and condition dependence of microstructure and carotenoids in yellow plumage coloration Proceedings of the Royal Society B: Biological Sciences 2006 273 2985 2991 10.1098/rspb.2006.3675 PMC1639519 17015356

[B51] Stuart-Fox DM Moussalli A Johnston GR Owens IP Evolution of color variation in dragon lizards: quantitative tests of the role of crypsis and local adaptation Evolution 2004 1549 1559 15341157 10.1111/j.0014-3820.2004.tb01735.x

[B52] Darst CR Cummings ME Cannatella DC A mechanism for diversity in warning signals: conspicuousness versus toxicity in poison frogs Proceedings of the National Academy of Sciences 2006 103 5852 10.1073/pnas.0600625103 PMC1458662 16574774

[B53] Stuart-Fox D Whiting MJ Moussalli A Camouflage and colour change: antipredator responses to bird and snake predators across multiple populations in a dwarf chameleon Biological Journal of the Linnean Society 2006 88 437 446 10.1111/j.1095-8312.2006.00631.x

[B54] Schaefer HM Levey DJ Schaefer V Avery ML The role of chromatic and achromatic signals for fruit detection by birds Behav Ecol 2006 17 784 789 10.1093/beheco/arl011

[B55] Newton I The Sparrowhawk 1986 A & C Black Publishers Ltd

[B56] Gosler AG Greenwood JJD Perrins C Predation risk and the cost of being fat Nature 1995 377 621 623 10.1038/377621a0

[B57] Götmark F Predation by sparrowhawks favours early breeding and small broods in great tits Oecologia 2002 130 25 32 10.1007/s004420100769 28547022

[B58] Geer TA Effects of Nesting Sparrowhawks on Nesting Tits The Condor 1978 80 419 422 10.2307/1367192

[B59] Dhondt A Kempenaers B Clobert J Sparrowhawk *Accipiter nisus *predation and blue tit *Parus caeruleus *adult annual survival rate Ibis 1998 140 580 584 10.1111/j.1474-919X.1998.tb04702.x

[B60] Vorobyev M Osorio D Bennett ATD Marshall NJ Cuthill IC Tetrachromacy, oil droplets and bird plumage colours Journal of Comparative Physiology A: Neuroethology, Sensory, Neural, and Behavioral Physiology 1998 183 621 633 10.1007/s003590050286 9839454

[B61] Håstad O Ödeen A Different Ranking of Avian Colors Predicted by Modeling of Retinal Function in Humans and Birds The American Naturalist 2008 171 831 838 10.1086/587529 18429674

[B62] Ödeen A Håstad O Complex Distribution of Avian Color Vision Systems Revealed by Sequencing the SWS1 Opsin from Total DNA Mol Biol Evol 2003 20 855 861 10.1093/molbev/msg108 12716987

[B63] Endler JA Mielke P Comparing entire colour patterns as birds see them Biological Journal of the Linnean Society 2005 86 405 431 10.1111/j.1095-8312.2005.00540.x

[B64] Stoddard M Prum R Evolution of Avian Plumage Color in a Tetrahedral Color Space: A Phylogenetic Analysis of New World Buntings Am Nat 2008 171 755 776 10.1086/587526 18419340

[B65] Hart NS Hunt DM Avian visual pigments: characteristics, spectral tuning, and evolution American Naturalist 2007 169 7 26 10.1086/510141 19426092

[B66] Lind O Kelber A Avian colour vision: effects of variation in receptor sensitivity and noise data on model predictions as compared to behavioural results Vision Res 2009 49 1939 1947 10.1016/j.visres.2009.05.003 19460402

[B67] v Campenhausen M Kirschfeld K Spectral sensitivity of the accessory optic system of the pigeon Journal of Comparative Physiology A: Neuroethology, Sensory, Neural, and Behavioral Physiology 1998 183 1 6 10.1007/s003590050229

[B68] Maier EJ Bowmaker JK Colour vision in the passeriform bird, *Leiothrix lutea*: correlation of visual pigment absorbance and oil droplet transmission with spectral sensitivity Journal of Comparative Physiology A: Neuroethology, Sensory, Neural, and Behavioral Physiology 1993 172 295 301 10.1007/BF00216611

[B69] Kelber A Vorobyev M Osorio D Animal colour vision: behavioural tests and physiological concepts Biological Reviews 2003 78 81 118 10.1017/S1464793102005985 12620062

[B70] Johnsen A Delhey K Andersson S Kempenaers B Plumage colour in nestling blue tits: sexual dichromatism, condition dependence and genetic effects Proceedings of the Royal Society B: Biological Sciences 2003 270 1263 1270 10.1098/rspb.2003.2375 PMC1691364 12816639

[B71] Andersson S Prager M Quantifying Colors Bird Coloration. Mechanisms and Measurements 2006 1 Cambridge, Massachusetts: Harvard University Press 41 89

[B72] Hart NS Partridge JC Cuthill IC Bennett ATD Visual pigments, oil droplets, ocular media and cone photoreceptor distribution in two species of passerine bird: the blue tit (*Parus caeruleus *L.) and the blackbird (*Turdus merula *L.) Journal of Comparative Physiology A: Neuroethology, Sensory, Neural, and Behavioral Physiology 2000 186 375 387 10.1007/s003590050437 10798725

[B73] Hart N Variations in cone photoreceptor abundance and the visual ecology of birds Journal of Comparative Physiology A: Neuroethology, Sensory, Neural, and Behavioral Physiology 2001 187 685 697 10.1007/s00359-001-0240-3 11778831

[B74] Håstad O Victorsson J Ödeen A Differences in color vision make passerines less conspicuous in the eyes of their predators Proceedings of the National Academy of Sciences of the United States of America 2005 102 6391 10.1073/pnas.0409228102 15851662 PMC1088363

[B75] Siddiqi A Cronin TW Loew ER Vorobyev M Summers K Interspecific and intraspecific views of color signals in the strawberry poison frog *Dendrobates pumilio* J Exp Biol 2004 207 2471 2485 10.1242/jeb.01047 15184519

[B76] Moyen F Gomez D Doutrelant C Pierson J Théry M Interacting effects of signalling behaviour, ambient light and plumage colour in a temperate bird, the blue tit *Parus caeruleus* Rev. Écol.(Terre Vie) 2006 61 367

[B77] Stevens M Cuthill IC Hidden Messages: Are Ultraviolet Signals a Special Channel in Avian Communication? BioScience 2007 57 501 10.1641/B570607

[B78] Cazetta E Schaefer H Galetti M Why are fruits colorful? The relative importance of achromatic and chromatic contrasts for detection by birds Evolutionary Ecology 2009 23 233 244 10.1007/s10682-007-9217-1

[B79] Avilés JM Vikan JR Fossøy F Antonov A Mosknes A Røskaft E Stokke BG Avian colour perception predicts behavioural responses to experimental brood parasitism in chaffinches Journal of Evolutionary Biology 2010 23 293 301 10.1111/j.1420-9101.2009.01898.x 20002251

[B80] Håstad O Victorsson J Ödeen A Differences in color vision make passerines less conspicuous in the eyes of their predators Proceedings of the National Academy of Sciences of the United States of America 2005 102 6391 6394 10.1073/pnas.0409228102 15851662 PMC1088363

[B81] Endler JA Natural selection on color patterns in *Poecilia reticulata* Evolution 1980 34 76 91 10.2307/2408316 28563214

[B82] Grether GF Hudon J Millie DF Carotenoid Limitation of Sexual Coloration along an Environmental Gradient in Guppies Proceedings: Biological Sciences 1999 266 1317 1322 10.1098/rspb.1999.0781

[B83] Hill GE Inouye CY Montgomerie R Dietary carotenoids predict plumage coloration in wild house finches Proceedings of the Royal Society B: Biological Sciences 2002 269 1119 1124 10.1098/rspb.2002.1980 PMC1691014 12061954

[B84] Hudon J Grether GF Millie DF Marginal differentiation between the sexual and general carotenoid pigmentation of guppies (*Poecilia reticulata*) and a possible visual explanation Physiological and Biochemical Zoology 2003 76 776 790 10.1086/378138 14988793

[B85] del Cerro S Merino S Martínez-de la Puente J Lobato E Ruiz-de-Castañeda R Rivero-de-Aguilar J Martínez J Morales J Tomás G Moreno J Carotenoid-based plumage colouration is associated with blood parasite richness and stress protein levels in blue tits (*Cyanistes caeruleus*) Oecologia 2010 162 825 835 10.1007/s00442-009-1510-y 19937348

[B86] Peters A Delhey K Denk A Kempenaers B Trade-offs between immune investment and sexual signaling in male mallards American Naturalist 2004 164 51 59 10.1086/421302 15266370

[B87] Ruxton G Sherratt T Speed M Avoiding Attack: The Evolutionary Ecology of Crypsis Warning Signals and Mimicry 2005 Oxford University Press

[B88] Arriero E Fargallo J Habitat structure is associated with the expression of carotenoid-based coloration in nestling blue tits *Parus caeruleus* Naturwissenschaften 2006 93 173 180 10.1007/s00114-006-0090-5 16508792

[B89] Isaksson C Andersson S Carotenoid diet and nestling provisioning in urban and rural great tits *Parus major* Journal of Avian Biology 2007 38 564 572

[B90] Isaksson C The chemical pathway of carotenoids: from plants to birds Ardea 2009 97 125 128

[B91] McGraw KJ Wakamatsu K Ito S Nolan PM Jouventin P Dobson FS Austic RE Safran RJ Siefferman LM Hill GE Parker RS You Can't Judge a Pigment by Its Color: Carotenoid and Melanin Content of Yellow and Brown Feathers in Swallows, Bluebirds, Penguins, and Domestic Chickens The Condor 2004 106 390 395 10.1650/7384

[B92] Trexler J Travis J Nontraditional Regression-Analyses Ecology 1993 74 1629 1637 10.2307/1939921

