# Supplementary material for: The carotenoid-continuum: carotenoid-based plumage ranges from conspicuous to cryptic and back again
Source: BMC Ecol. 2010 May 26;10:13. doi: 10.1186/1472-6785-10-13 (PMC2896926; doi:10.1186/1472-6785-10-13)
Supplement: Additional file 1 — Plumage Reflectance Spectra of blue tits (Figure 1), great tits (Figure 2) and greenfinches (Figure 3). Depicted are mean reflectance spectra for males, females and fledglings and treatment groups (blue tit only). [file 1472-6785-10-13-S1.PDF]

Additional file 1 - Plumage reflectance spectra of blue tits (Fig. 1), great tits (Fig. 2) and greenfinches (Fig. 3). Depicted are mean reflectance spectra for males, females and fledglings and for experimental treatment groups (blue tit only).

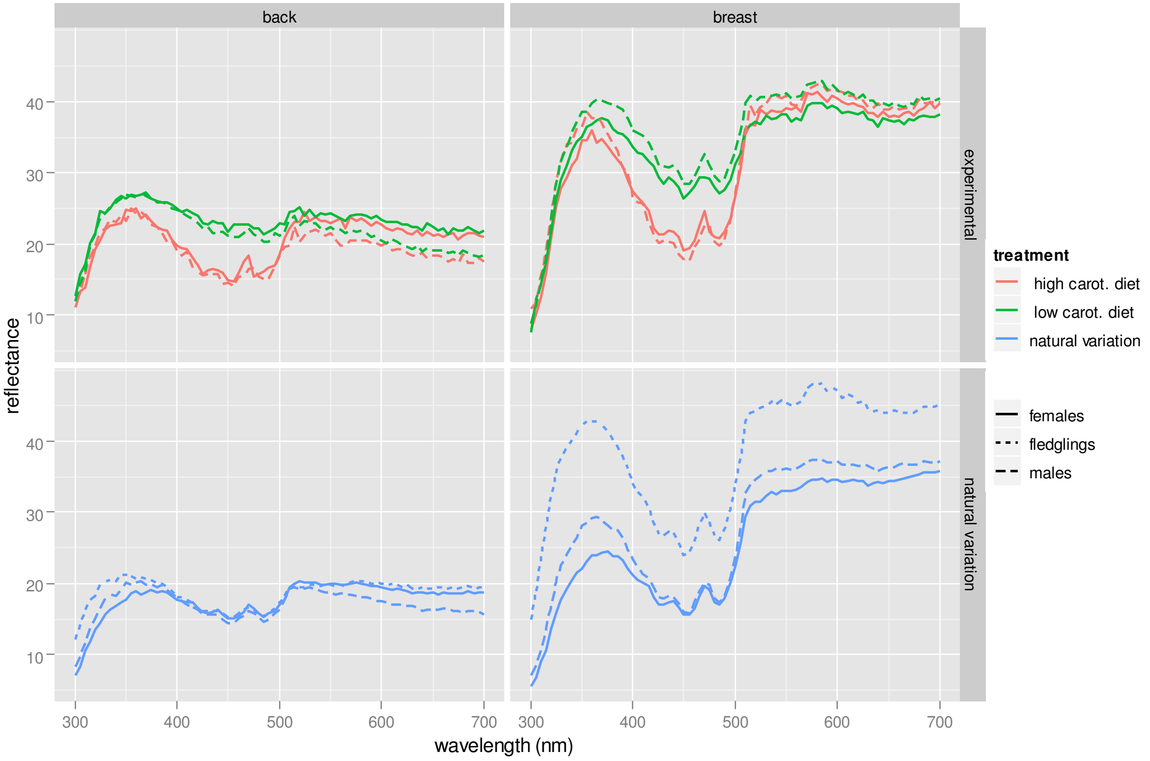

Figure 1

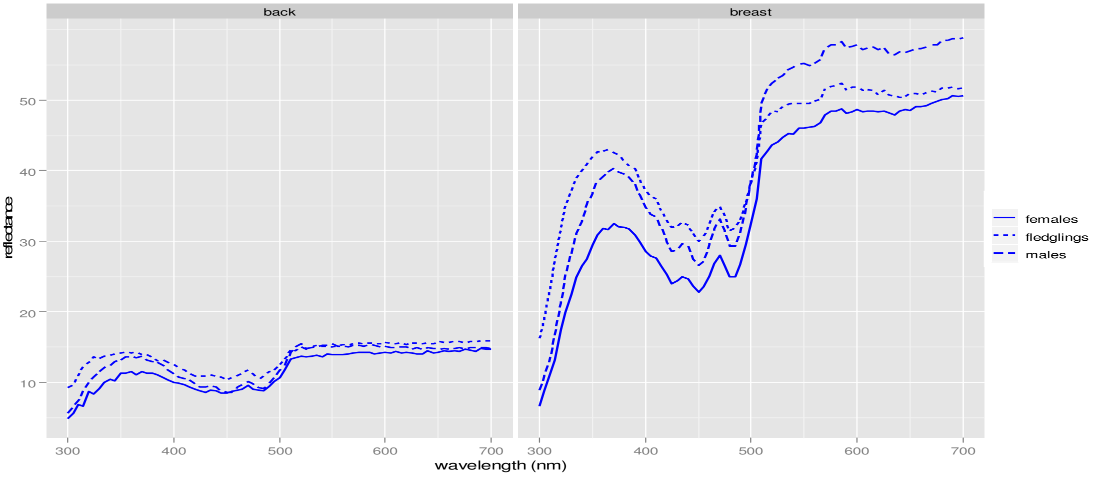

Figure 2

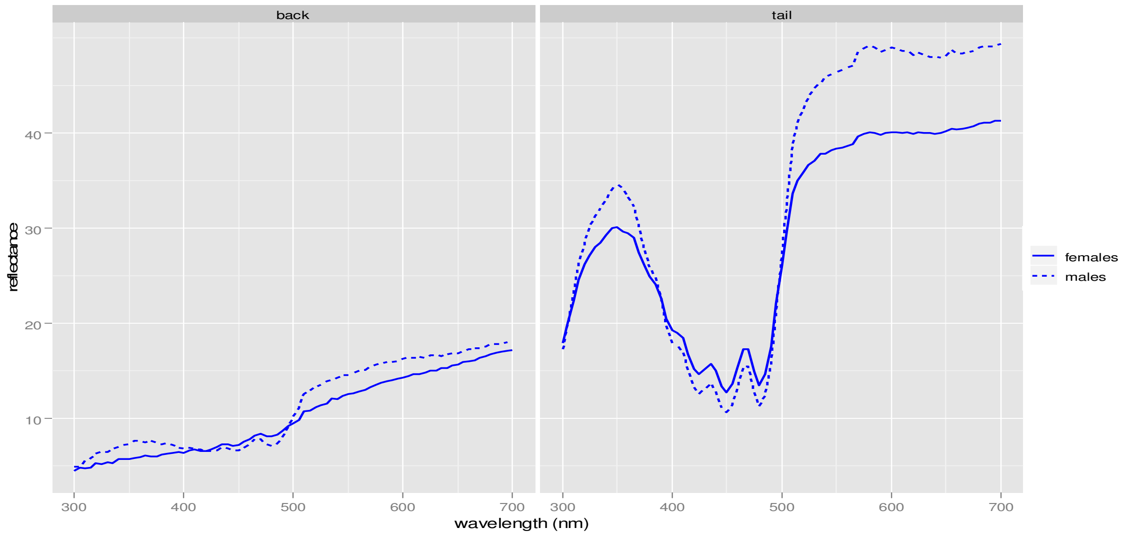
back
tail
reflectance
40
30
20
10
300
400
500
600
700
wavelength (nm)
females
males

Figure 3
