# Supplementary material for: The carotenoid-continuum: carotenoid-based plumage ranges from conspicuous to cryptic and back again
Source: BMC Ecol. 2010 May 26;10:13. doi: 10.1186/1472-6785-10-13 (PMC2896926; doi:10.1186/1472-6785-10-13)
Supplement: Additional file 2 — List of common plants in the study area (Table 1, including relative abundance and number of collected reflectance spectra (N)) and their reflectance spectra (Figure 1). Depicted are mean reflectance spectra for brown (branches, twigs, leaf-litter) and green (leaves) backgrounds. Red lines represent mean spectra for each plant type separately and blue lines the overall brown and green averages taking into account the abundance of each plant type. [file 1472-6785-10-13-S2.PDF]

Additional file 2 – List of common plants in the study area (Table 1, including relative abundance and number of collected reflectance spectra (N)) and their reflectance spectra (Fig. 1). Depicted are mean reflectance spectra for brown (branches, twigs, leaf-litter) and green (leaves) backgrounds. Red lines represent mean spectra for each plant type separately and blue lines the overall brown and green averages taking into account the abundance of each plant type.

Table 1. List of common plants in the study area; including relative abundance and number of collected reflectance spectra (N). The number (Nr.) acts as a reference for the figures in Additional Files 4.

**Nr.**	Plant	relative abundance[1]	N leaves	N bark, branches and twigs	N leaf litter
**1**	*Acer pseudoplatanus*	8	60	111	
**2**	*Malus domestica*	1	30	51	
**3**	*Rubus sp.*	1	10	5	
**4**	*Berberis julianae*	1	20	40	
**5**	*Betula pendula*	1	10	20	
**6**	*Fagus sylvatica*	5	80	145	10
**7**	*Hedera helix*	8	40	20	
**8**	*Quercus robur*	7	20	50	20
**9**	*Fraxinus excelsior*	7	10	30	
**10**	*Picea abies*	6	10	15	
**11**	*Syringa vulgaris*	1	30	35	
**12**	*Myrica gale*	1	10	20	
**13**	*Corylus colurna*	7	20	35	
**14**	*Carpinus betulus*	1	20	40	
**15**	*Lonicera xylosteum*	1	40	61	
**16**	*Sambucus nigra*	6	70	105	
**17**	*Aesculus hippocastanum*	1	30	55	
**18**	*Thuja occidentalis*	1	10	20	
**19**	*Ligustrum vulgare*	1	20	30	
**20**	*Tilia platyphyllos*	1	10	20	
**21**	*Rosa sp.*	1	50	80	
**22**	*Phragmites sp.*	1	10	10	
**23**	*Viburnum lantana*	1	30	50	
**24**	*Prunus padus*	4	50	70	
**25**	*Salix sp.*	2	30	51	
**26**	unidentif. grasses	1	15		20

[1]arbitrary scale based on 10 point counts along a transect in the study area.

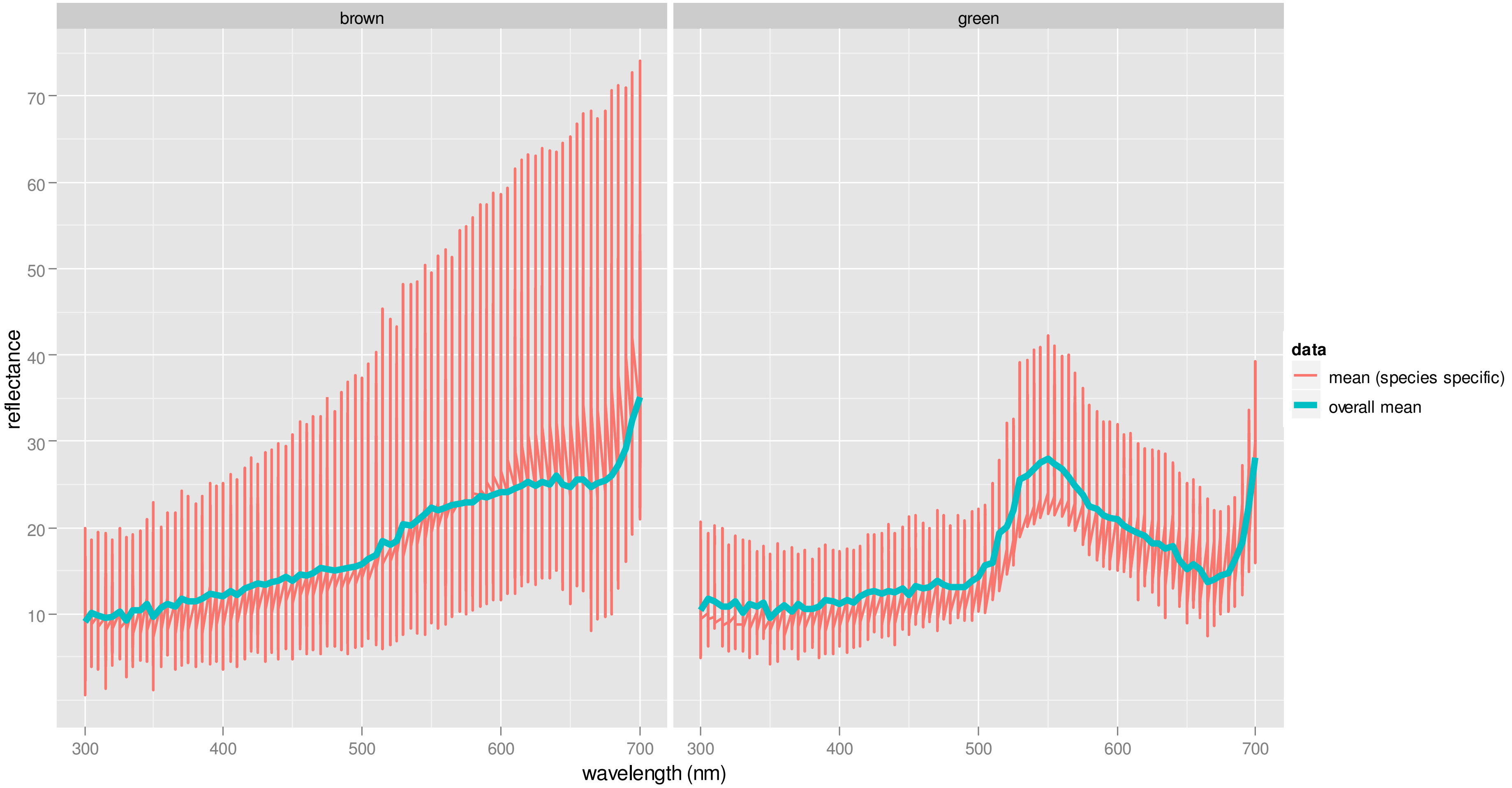
brown
green
reflectance
70
60
50
40
30
20
10
300
400
500
600
700
wavelength (nm)
data
mean (species specific)
overall mean

Figure 1
