# Supplementary material for: The carotenoid-continuum: carotenoid-based plumage ranges from conspicuous to cryptic and back again
Source: BMC Ecol. 2010 May 26;10:13. doi: 10.1186/1472-6785-10-13 (PMC2896926; doi:10.1186/1472-6785-10-13)
Supplement: Additional file 3 — Effects of inter-specific variation in cone proportions [file 1472-6785-10-13-S3.PDF]

Additional file 3 – Effects of inter-specific variation in cone proportions

Retinal cone proportions are required by the Vorobyev-Osorio model to estimate the signal-to-noise ratio of each cone type [1], and variation in this parameter can have considerable effects on calculated discriminability or contrast [2]. As the retinal cone proportions of great tits, greenfinches (which have U-type eyes) and birds of prey (which have V-type eyes) are unknown we assess how variation in this parameter affects our results. To this effect we use the data of Hart [3] on the inter-specific variation in retinal cone proportions of 22 bird species.

We repeated the computations using data from all 22 species to assess whether it affects the relationship between chroma and contrast. The general curvilinear relationship between chroma and contrast against green backgrounds was not affected by the use of different retinal cone proportions (the conclusions are the same if we use brown backgrounds). In all cases but one, yellow and green plumages were more contrasting to U-type eyes with blue tit cone proportions than to U- or V-type eyes with the cone proportions of the other species modelled here (Figs 1, 2). The exception was using cone proportions of the pied cormorant *Phalacrocorax varius* which yielded higher plumage contrast against natural backgrounds especially at high values of chroma (Fig. 2).

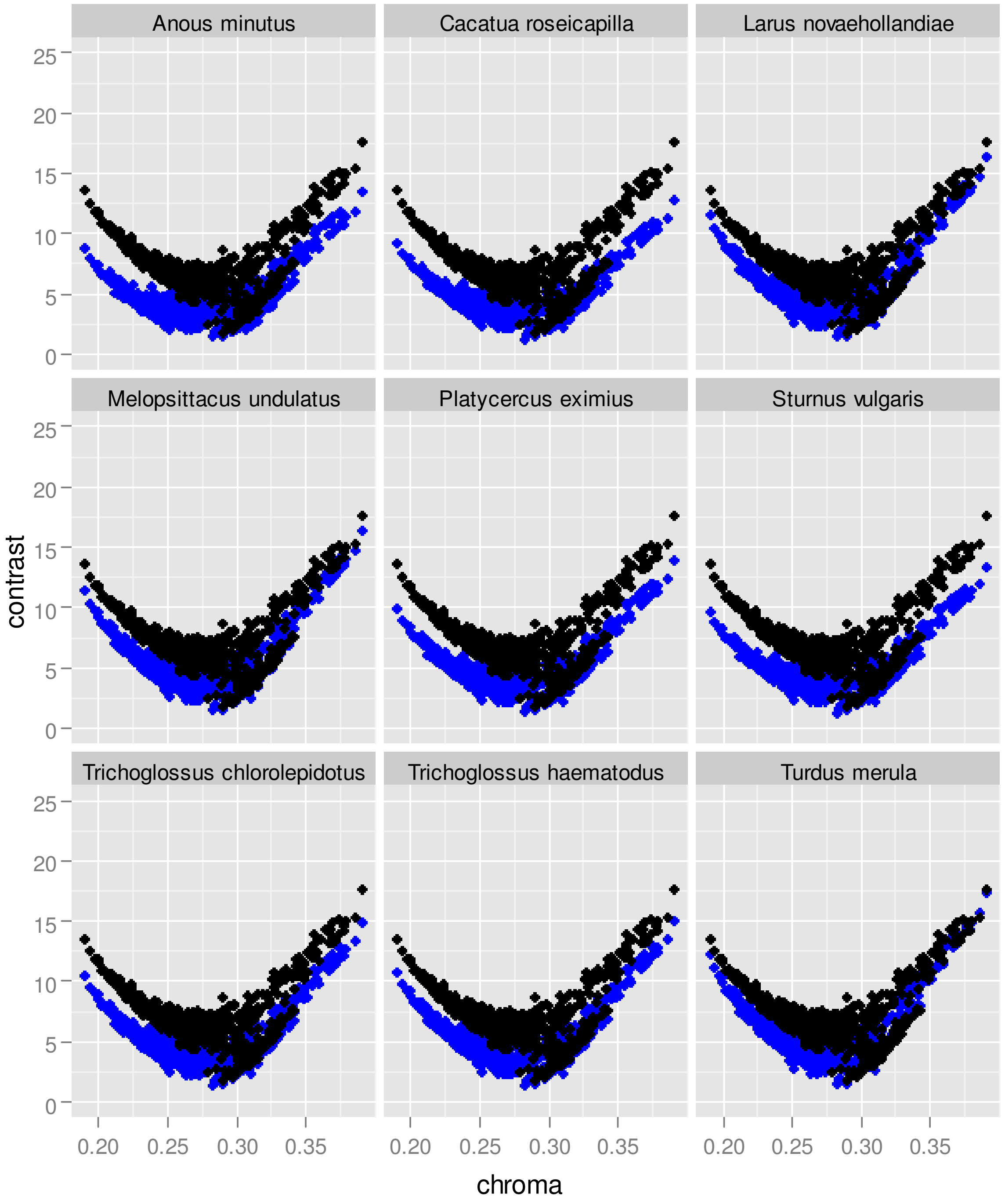

Figure 1. Relationship between carotenoid-based plumage coloration chroma (pooling green and yellow plumage patches of all three study species) and contrast against average green backgrounds comparing U-type eyes with blue tit cone proportions (black dots) against U-type eyes with cone proportions belonging to 9 species with U-type eyes (blue dots) listed in Table 1 (data from [3]).

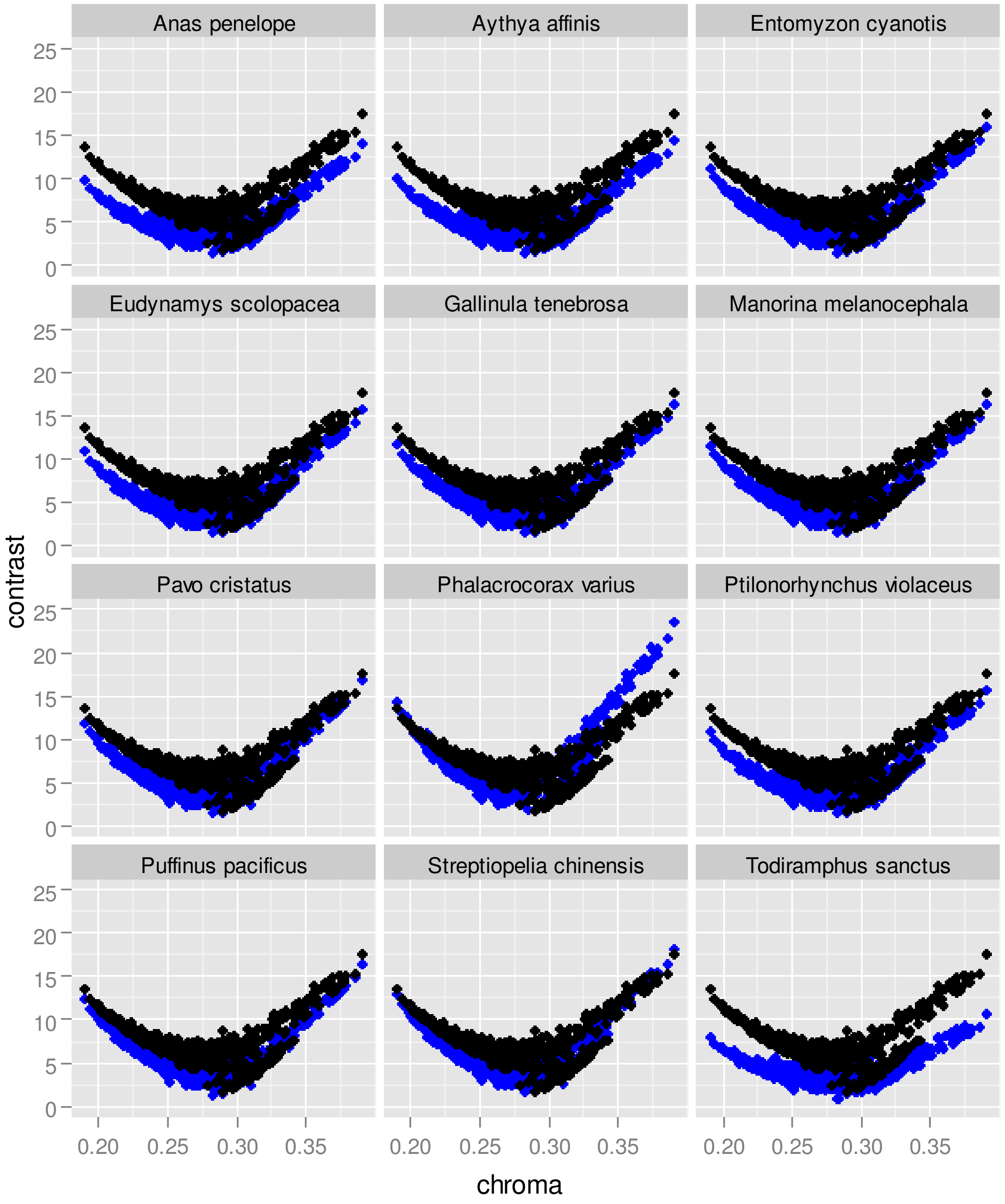

Figure 2. Relationship between carotenoid-based plumage coloration chroma (pooling green and yellow plumage patches of all three study species) and contrast against average green backgrounds comparing U-type eyes with blue tit cone proportions (black dots) against V-type eyes with cone proportions belonging to 12 species with V-type eyes (blue dots) listed in Table 1 (data from [3]).

Table 1. Proportions of single cones in the retinas of 22 bird species studied by Hart [3] expressed as fraction of the L cone abundance.

**Common name**	**Latin name**	**eye type**[1]	**VS**	**S**	**M**	**L**
Lesser Scaup	*Aythya affinis*	V-type	0.24	0.65	0.97	1
Wigeon	*Anas penelope*	V-type	0.24	0.50	1.02	1
Black Noddy	*Anous minutus*	UV-type	0.07	0.67	1.18	1
Silver Gull	*Larus novaehollandiae*	UV-type	0.43	0.80	1.02	1
Spotted Turtle Dove	*Streptiopelia chinensis*	V-type	0.70	0.87	1.12	1
Sacred Kingfisher	*Todiramphus sanctus*	V-type	0.16	0.21	0.24	1
Common Koel	*Eudynamys scolopacea*	V-type	0.32	0.73	1.17	1
Peafowl	*Pavo cristatus*	V-type	0.47	0.89	1.04	1
Dusky Moorhen	*Gallinula tenebrosa*	V-type	0.46	0.77	0.96	1
Blue-faced Honeyeater	*Entomyzon cyanotis*	V-type	0.38	0.75	1.03	1
Noisy Miner	*Manorina melanocephala*	V-type	0.43	0.80	0.98	1
Satin Bowerbird	*Ptilonorhynchus violaceus*	V-type	0.32	0.75	1.12	1
European Starling	*Sturnus vulgaris*	UV-type	0.27	0.36	0.98	1
Blackbird	*Turdus merula*	UV-type	0.53	0.90	1.13	1
Blue tit	*Cyanistes caeruleus*	UV-type	0.37	0.71	1.00	1
Pied Cormorant	*Phalacrocorax varius*	V-type	0.70	1.72	4.09	1
Wedge-tailed Shearwater	*Puffinus pacificus*	V-type	0.69	0.47	0.72	1
Galah	*Cacatua roseicapilla*	UV-type	0.24	0.30	0.95	1
Budgerigar	*Melopsittacus undulatus*	UV-type	0.40	0.76	1.19	1
White-cheeked Rosella	*Platycercus eximius*	UV-type	0.26	0.49	0.93	1
Scaly-breasted Lorikeet	*Trichoglossus chlorolepidotus*	UV-type	0.32	0.56	1.06	1
Rainbow Lorikeet	*Trichoglossus haematodus*	UV-type	0.38	0.49	1.09	1

[1]kindly provided by Anders Ödeen

References

1. Vorobyev M, Osorio D: **Receptor noise as a determinant of colour thresholds.** *Proceedings of the Royal Society B: Biological Sciences* 1998, **265**:351.

2. Lind O, Kelber A: **Avian colour vision: effects of variation in receptor sensitivity and noise data on model predictions as compared to behavioural results**. *Vision Res* 2009, **49**:1939-1947.

3. Hart N: **Variations in cone photoreceptor abundance and the visual ecology of birds**. *Journal of Comparative Physiology A: Neuroethology, Sensory, Neural, and Behavioral Physiology* 2001, **187**:685-697.
