# Supplementary material for: The carotenoid-continuum: carotenoid-based plumage ranges from conspicuous to cryptic and back again
Source: BMC Ecol. 2010 May 26;10:13. doi: 10.1186/1472-6785-10-13 (PMC2896926; doi:10.1186/1472-6785-10-13)
Supplement: Additional file 4 — Figures representing the relationship between chroma and detectability for each type of background depicting raw data points. [file 1472-6785-10-13-S4.PDF]

Additional file 4 – Relationship between chroma and contrast against each different type of background in the study site as seen through V-type eyes (the relationship for U-type eyes is very similar). Brown backgrounds are depicted in Fig. 1 and green backgrounds in Fig. 2. Numbers correspond to reference numbers in Table 1 of Additional File 2 except for 27, 28 and 29 which correspond to grass, beech and oak leaf-litter respectively.

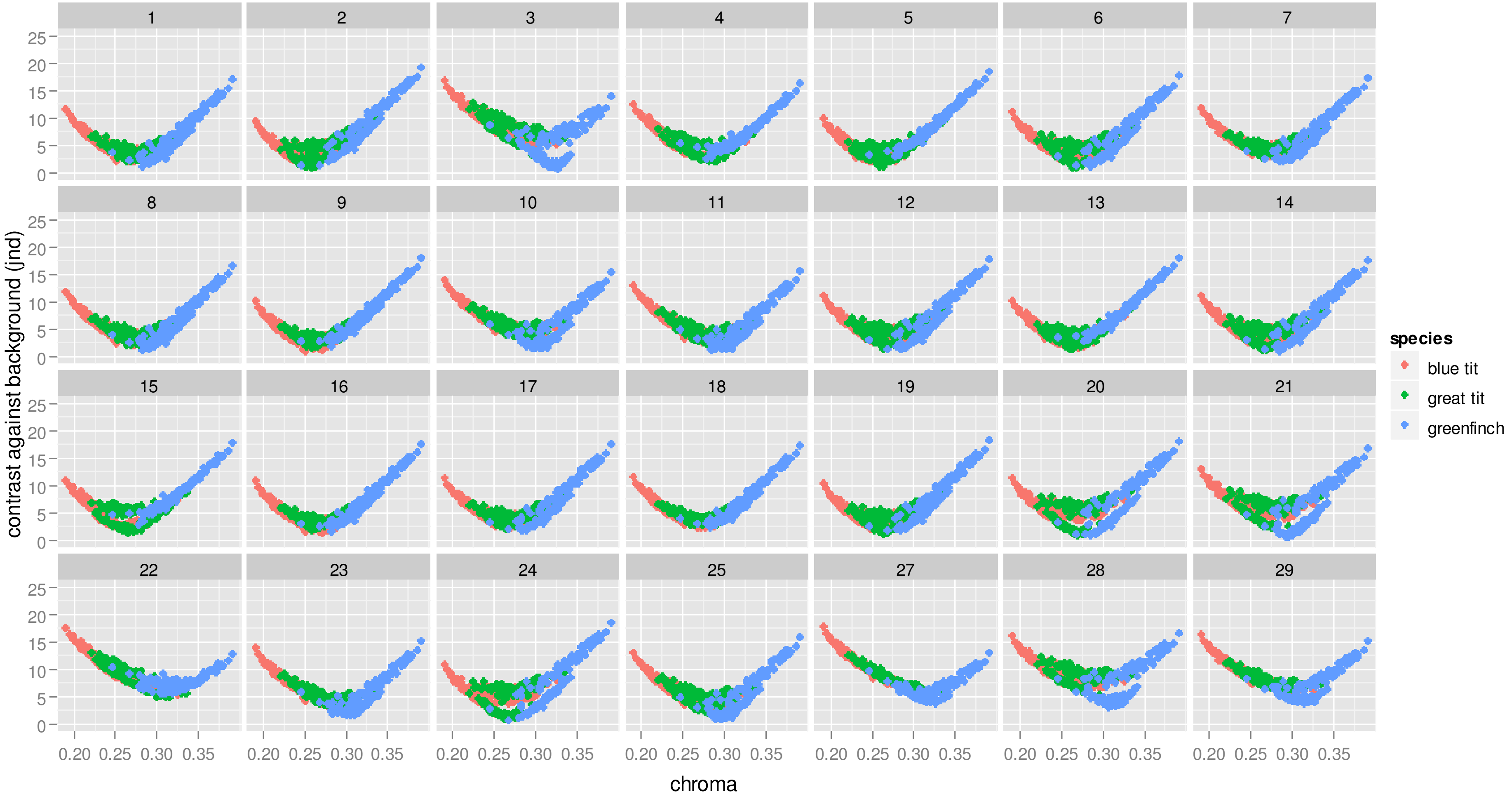

Figure 1

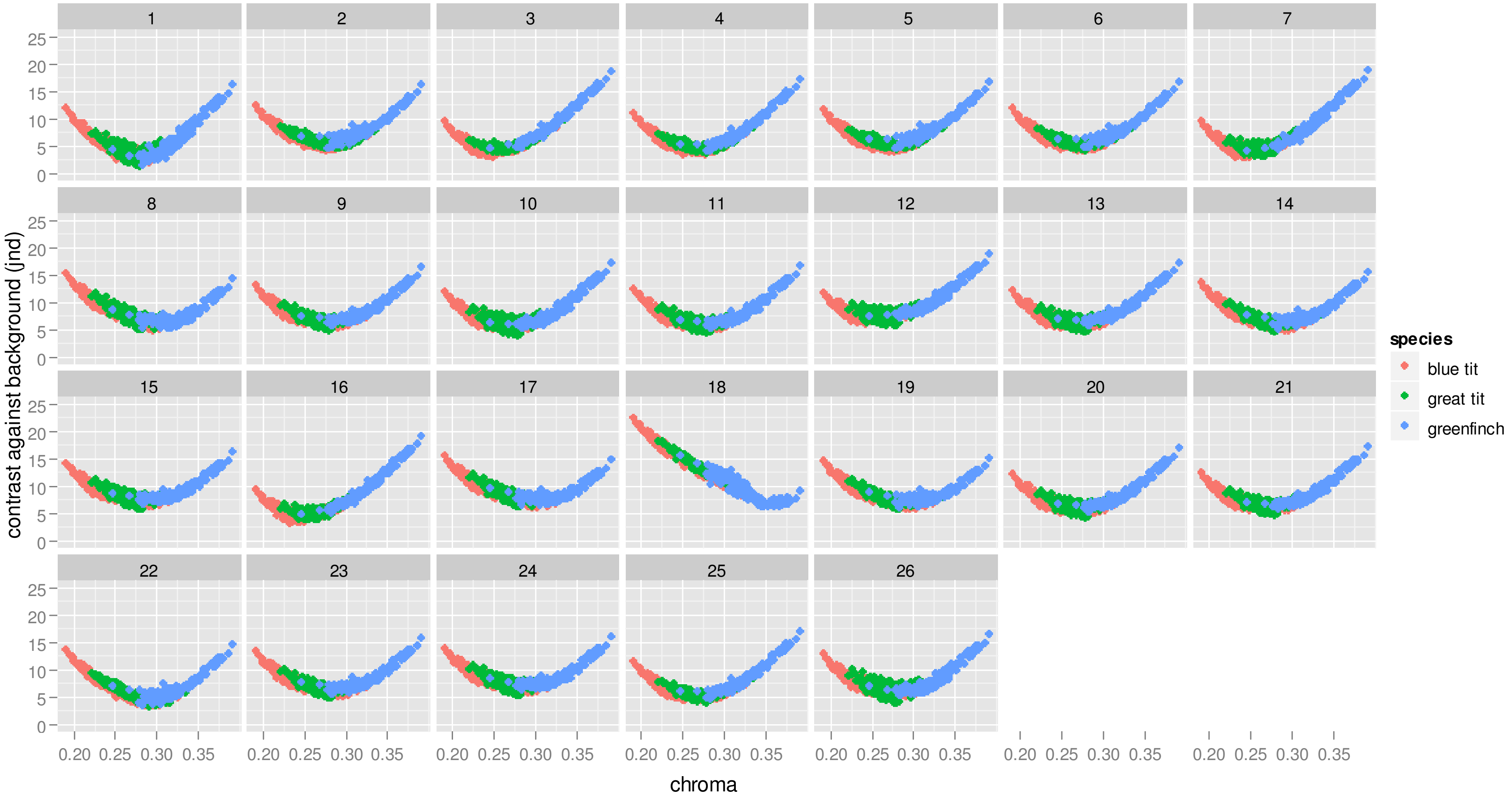

1
2
3
4
5
6
7
8
9
10
11
12
13
14
15
16
17
18
19
20
21
22
23
24
25
26
25
20
15
10
5
0
contrast against background (jnd)
0.20 0.25 0.30 0.35
chroma
species
blue tit
great tit
greenfinch

Figure 2
